# Intranasal delivery of a recombinant adenovirus vaccine encoding the PEDV COE elicits potent mucosal and systemic antibody responses in mice

**DOI:** 10.1128/spectrum.00692-24

**Published:** 2024-08-15

**Authors:** Shijie Yan, Yi Luo, Ningjia Zhan, Haoran Xu, Yao Yao, Xiang Liu, Xiaoqing Dong, Li Kang, Guozhong Zhang, Pinghuang Liu

**Affiliations:** 1 National Key Laboratory of Veterinary Public Health and Safety, College of Veterinary Medicine, China Agricultural University, Beijing, China; 2 Key Laboratory of Animal Epidemiology of Ministry of Agriculture and Rural Affairs, College of Veterinary Medicine, China Agricultural University, Beijing, China; Changchun Veterinary Research Institute, Changchun, China

**Keywords:** PEDV, vaccine, Ad5 vector, intranasal immunization, mucosal immune responses

## Abstract

Porcine epidemic diarrhea virus (PEDV) is an enteropathogenic coronavirus that causes substantial economic loss to the global pig industry. The emergence of PEDV variants has increased the need for new vaccines, as commercial vaccines confer inferior protection against currently circulating strains. It is well established that the induction of mucosal immunity is crucial for PEDV vaccines to provide better protection against PEDV infection. In this study, we constructed a recombinant adenovirus expressing the core neutralization epitope (COE) of G2b PEDV based on human adenovirus serotype 5 (Ad5). We evaluated the effects of different administration routes and doses of vaccine immunogenicity in Balb/c mice. Both intramuscular (IM) and intranasal (IN) administration elicited significant humoral responses, including COE-specific IgG in serum and mucosal secretions, along with serum-neutralizing antibodies. Moreover, IN delivery was more potent than IM in stimulating IgA in serum and mucosal samples and in dampening the immune response to the Ad5 vector. The immune response was stronger after high versus low dose IM injection, whereas no significant difference was observed between high and low IN doses. In summary, our findings provide important insights for developing novel PEDV vaccines.

**IMPORTANCE:**

Porcine epidemic diarrhea (PED) is a highly contagious disease that has severe economic implications for the pork industry. Developing an effective vaccine against PEDV remains a necessity. Here, we generated a recombinant adenovirus vaccine based on Ad5 to express the COE protein of PEDV (rAd5-PEDV-COE) and systematically evaluated the immunogenicity of the adenovirus-vectored vaccine using different administration routes (intramuscular and intranasal) and doses in a mouse model. Our results show that rAd5-PEDV-COE induced potent systemic humoral response regardless of the dose or immunization route. Notably, intranasal delivery was superior to induce peripheral and mucosal IgA antibodies compared with intramuscular injection. Our data provide valuable insights into designing novel PEDV vaccines.

## INTRODUCTION

Porcine epidemic diarrhea (PED) is an acute, highly contagious disease characterized by acute gastroenteritis, diarrhea, vomiting, and dehydration (1). Pigs of all ages are susceptible to the pathogen, exhibiting different clinical symptoms. Mortality due to viral infection varies with age, with up to 100% mortality in 1–3-day-old piglets (2). In 2010, a new variant PEDV strain emerged in China for the first time, causing enormous economic losses in the pork industry (3, 4). PED first emerged in 2013 in the United States and quickly spread throughout the country and caused a high number of pig deaths (5). PEDV is now a global pathogen, spreading through pig herds worldwide. The frequent outbreaks of PEDV even in vaccinated pork farms indicate compelling needs to develop better new vaccines against PEDV infection. Given that PEDV primarily infects the small intestines and causes serious gastrointestinal damage *in vivo*, it is well established that the induction of mucosal immunity, especially gastrointestinal mucosal immunity, is crucial for PEDV vaccines to provide effective protection against PEDV infection.

PEDV is an enveloped, positive single-strand RNA virus that belongs to genus *Alphacoronavirus*, family Coronaviridae. The whole genome consists of seven open reading frames (ORFs) encoding 16 accessory proteins and four structural proteins, such as spike glycoprotein (S), membrane protein (M), envelope protein (E), and nucleocapsid protein (N) (6). Spike protein is responsible for cell attachment and virus–host membrane fusion mediating viral entry into host cells, and as the major surface glycoprotein on the enveloped virions, the core neutralization epitope (COE) region of coronavirus S protein is responsible for recognizing and binding cellular targets, and is an important target of the host antibody response and a critical candidate for the development of the coronavirus vaccine (7, 8).

Adenovirus vectors are the most commonly used vector for vaccine development due to their integrating foreign gene expression and transduction capabilities (9, 10). The most widely used adenoviral vector is the replication-incompetent human adenovirus five vector (Ad5), owing to its significant advantages in safety and inducing adaptive and mucosal immune responses (11–13). For example, the CanSino vaccine, designed based on Ad5, has been administered to millions of people worldwide and has shown potent protection against SARS-CoV-2 (14). Additionally, Ad5 can infect various porcine cell lines, such as ST and IPEC, and replicate efficiently in porcine intestine and mesenteric lymph nodes (15), and these characteristics make Ad5 vectors widely utilized in vaccine research and development. In this study, we generated a recombinant adenovirus vaccine based on Ad5 to express the COE protein of PEDV (rAd5-PEDV-COE) and evaluated the immune responses of this vaccine in mice. Our findings provide a strong foundation for developing a novel vaccine against PEDV.

## MATERIALS AND METHODS

### Cells, viruses, and plasmids

HEK 293 cells (human embryonic kidney, Chinese National Collection of Authenticated Cell Cultures) and Vero cells (African green monkey kidney, ATCC, China) were grown and maintained in DMEM (Gibco, Cat.11995500BT) supplemented with antibiotics (100 µg/mL streptomycin and 100 units/mL penicillin) and 10% heat-inactivated fetal bovine serum (Gibco, Cat.10091148), and both cultures were maintained at 37°C and 5% CO_2_. The field-isolated strain G2b PEDV strain JMS (GenBank No. PP461398.1) and PEDV strain CV777 (GenBank No. KT323979) stocked in our laboratory were grown and passaged in Vero cells described in our previous study (16–18).

### Construction and preparation of recombinant adenovirus expressing PEDV S protein COE

The recombinant adenovirus was constructed and produced based on the handbook of the AdEasy Adenoviral Vector System (Agilent, CA). In brief, PEDV-COE sequence spike 219–729 gene based on PEDV-JMS spike (GenBank No. PP461398) was codon optimized by GenScript (Nanjing, China) and cloned into Pshuttle-CMV vector through the endonucleases KpnI and XhoI. Then, the resulting shuttle plasmid Pshuttle-CMV-PEDV-COE was transformed into *Escherichia coli* BJ5183-AD-1 containing the adenoviral genome backbone pAdEasy-1 (Weidibio, China) by homologous recombination to construct recombinant adenoviral plasmid. The linearized recombinant adenoviral DNA was transfected into HEK293 cells by using Lipofectamine 3000 (Invitrogen, Cat.L3000015) to produce recombinant adenoviruses. The cells and supernatant were harvested when 70%–80% cytopathic effect was observed, then three freeze–thaw–vortex cycles were performed to release adenovirus and centrifuged at 10,000 *g* at 4°C to pellet the cell debris. The particle number and infectivity of the recombinant adenovirus were determined by optical density and immunodetection of the hexon protein of AdHu5 (Adeno-X rapid titer kit; Cat. 632250 Clontech) according to the manufacturer’s instructions.

### Identification of PEDV-COE expression by ELISA

To evaluate the expression of COE by rAd5-PEDV-COE, HEK293 cells in cell 24-well culture plates were infected with rAd5-PEDV-COE at a multiplicity of infection (MOI) of 2. After 48 h postinfection, the supernatant was collected. The anti-COE antibodies were measured by an in-house anti-COE enzyme-linked immunosorbent assay (ELISA). Briefly, 96-well plates (Corning, NY) were coated with 100 µL of 1 µg/mL COE expression supernatant in 50-mM carbonate buffer (pH 9.6) at 4°C overnight. The plates were washed three times with phosphate-buffered saline (PBS), pH 7.4, containing 0.05% Tween-20 (PBST) and then blocked with blocking buffer (PBST with 5% skim milk and 5% FBS) for 2 h at 37°C. After washing, the mouse anti-COE monoclonal antibody (prepared by our laboratory) was added in duplicate for 1 h at 37°C. After washing, horseradish peroxidase (HRP)-conjugated goat anti-mouse IgG Fc (Abcam, Cat.ab97265) diluted 1:20,000 was added and incubated for 1 h. After washing by PBST, the tetramethylbenzidine (TMB) peroxidase substrate with H_2_O_2_ was added and incubated at 37°C for 10 min. The reaction was stopped with 50 µL 2M H_2_SO_4_. The optical density (OD) was measured with a Bio-Tek microplate reader at 450 nm.

### Identification of PEDV-COE expression by immunofluorescence assay (IFA)

To determine the expression of COE by rAd5-PEDV-COE, HEK293 cells in 48-well culture plates were infected with rAd5-PEDV-COE at a MOI of 0.5. The plates were then incubated for 36 h at 37°C in a 5% CO_2_ and analyzed by IFA subsequently. The cells were fixed with 4% paraformaldehyde for 30 min, then were permeabilized with 0.2% Triton X-100 for 20 min, and blocked for 2 h at 37°C. The cells were stained with 100-µL mouse anti-COE monoclonal antibody (prepared by our laboratory). The bound antibodies were visualized using the 500-fold dilution Goat anti-Mouse IgG (H + L)-Alexa Fluor 546 (Invitrogen, Cat.A-11003) for 1 h at 37°C. Cell nuclei were counterstained with DAPI (1 µg/mL) (Sigma-Aldrich, St. Louis, MO). The plates were examined, and images were captured under the Nikon inverted fluorescence microscope.

### Vaccination of the Ad5-COErAd5-PEDV-COE in mice

Forty-eight specific pathogen-free (SPF) female Balb/c mice aged 6–8 weeks from Charles River (Beijing, China) were randomly divided into six groups. The mice were immunized intramuscularly (IM) or intranasally (IN) with different doses of rAd5-PEDV-COE, and the immune dose was determined by reference to the research performed by Sanchez et al. (19) and made some modifications. Specifically, the mice in the IM-high dose (HD) and IN-HD groups received a prime with 10^8^ PFU and then a boost with 10^8^ PFU; the mice in the IM-low dose (LD) group received a prime with 10^6^ PFU and then a boost with 10^8^ PFU; and the mice in the IN-LD group received a prime with 10^7^ PFU and then a boost with 10^7^ PFU. The empty vector group was immunized intranasally with 10^8^ PFU/mouse of the Ad5 vector, and the mock group was immunized with 1 mL of PBS via IM injection. A second boost immunization was performed 10 weeks after initial immunization, and the immune dose and pathway were the same as that of the first boost immunization. Serum and mucosal samples were collected from all mice at the indicated time points. The collection and treatment of mucosal samples were described as previously published (20). Specifically, the mice were euthanized, and an injection syringe was inserted gently into the lumen of the exposed trachea. The lungs were douched with sterile normal saline, and the lavage fluid was then collected and stored at −80°C until examination. As for intestinal contents, the contents were collected and re-mixed with PBS containing 0.1% BSA and centrifuged for 5 min at 4°C at 3000 *g*. Supernatants were collected and stored at −80°C until testing. The anti-COE antibodies in samples were evaluated by indirect ELISA or microplate neutralization assay. All mice experiments were performed in strict accordance with the Guide for the Care and Use of Laboratory Animals of the People’s Republic of China and were approved by the China Agriculture University Institutional Animal Care and Use Committee.

### COE-specific binding IgG or IgA antibodies detection by ELISA

An indirect ELISA based on PEDV-COE protein was developed to detect anti-PEDV IgG and IgA antibodies in the immunological serum, intestinal contents, and BALFs. ELISA plates were coated with 1 µg/mL COE protein at 4°C overnight. Then, the plates were blocked with 5% skim milk and 5% FBS at 37°C for 2 h, and diluted sera or intestinal lavage fluids (1:8 dilution) or bronchoalveolar fluid (1:4 dilution) were added to the plates and incubated at 37°C for 90 min. HRP-conjugated goat anti-mouse IgG antibody (1:20,000 dilution) or HRP-conjugated goat anti-mouse IgA antibody (1:4,000 dilution) was added and incubated at 37°C for 1 h followed by addition and incubation in TMB substrate for 10 min after washing. Absorbance values were determined at 450 nm after the addition of 50-µL 2M H_2_SO_4_ stop solution. The cut-off value was determined by calculating the mean OD450nm＋3× standard derivations (SD) from the serum of nonvaccinated mice. The endpoint titers of the serum were then calculated as the reciprocal of the highest serum dilution at which the OD_450nm_ values were equal to or greater than the cut-off value. Data analysis was conducted using GraphPad Prism 8.0.

### PEDV microplate neutralization assay

Neutralizing antibodies of serum were determined using microplate neutralization assay as previously described (21). Vero cells were seeded in 96-well plates and cultured overnight to form a confluent monolayer. Subsequently, 400 TCID_50_ of PEDV-JMS was mixed with an equal volume of a twofold serial dilution of serum and incubated at 37°C for 1 h, followed by the inoculation onto Vero cells at 37°C for 2 h. After the virus–serum mixture was removed, the cells were washed gently with DMEM thrice, maintained in a medium containing 3‰ trypsin. The plates were then incubated for 30 h at 37°C in a 5% CO_2_ atmosphere, and PEDV infection was evaluated by PEDV nucleocapsid IFA assay. The fluorescence of each well was recorded under the microscopes, and the neutralizing titer was the reciprocal of the highest dilution that showed 80% reduction in the fluorescence compared with controls, and the NAb titer was calculated based on these data. The neutralization assay for PEDV-CV777 is the same as that for PEDV-JMS mentioned above, and serum dilution was 1:160 and 1:640.

### Statistical analysis

All data analyses and graph plotting were performed using GraphPad Prism 8.0. The anti-PEDV-COE IgG and IgA titers and NAbs of all samples were compared by correlation analysis, and the values were presented as correlation and mean ± standard deviation (SD). R^2^ values represent correlation coefficients, and *P* < 0.05 was considered statistically significant. The differences between groups were compared using an unpaired *t*-test. Differences were considered significant if the *P*-value was < 0.05, and the *P*-values were denoted as follows: ∗*P* < 0.05; ∗∗*P* < 0.01; ∗∗∗*P* < 0.001; ∗∗∗∗*P* < 0.0001.

## RESULTS

### Construction and validation of recombinant adenovirus RAd5-PEDV-COE plasmid

**Fig 1:**
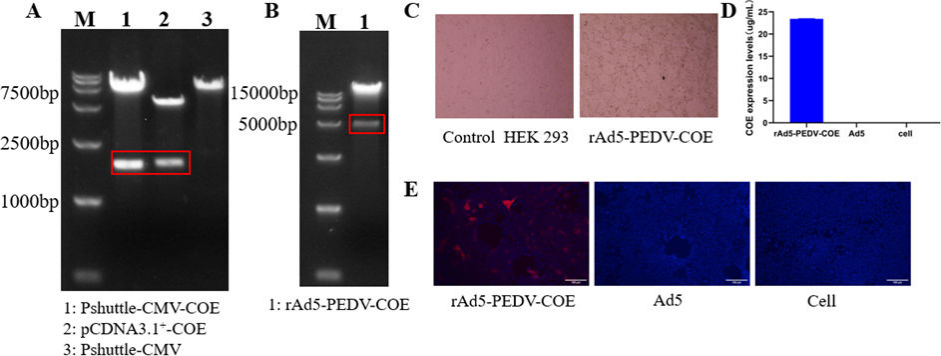
Construction and characterization of recombinant adenovirus 5 (Ad5) vector expressing PEDV-COE. (**A**) Restriction enzyme digestion of Pshuttle-CMV-COE recombinant plasmid. (**B**) Construction of rAd5-PEDV-COE. (**C**) Rescue of rAd5-PEDV-COE. (**D**) ELISA verification of rAd5-PEDV-COE. (**E**) Detection of PEDV COE expression in HEK 293 cells infected with rAd5-PEDV-COE.

The optimized PEDV-COE gene was cloned into the Pshuttle-CMV plasmid, and the results showed that the target gene bands of the Pshuttle-CMV-COE plasmid were the same as those of the pCDNA3.1-COE plasmid, which indicated that the construction of the Pshuttle-CMV-COE plasmid was successful (Fig. 1A). The Pshuttle-CMV-COE plasmid was homologously recombined with the Ad5 backbone plasmid, and the recombinant Ad5 plasmid was verified by the PacI digestion results that the rAd5-PEDV-COE plasmid had a band at 4500 bp, indicating that the recombinant rAd5-PEDV-COE plasmid was successfully constructed (Fig. 1B). The rAd5-PEDV-COE adenovirus was prepared by transfecting HEK293 cells, which showed obvious cytopathic lesions 10 days after transfection, whereas no cytopathic lesions were found in the control wells (Fig. 1C). Subsequently, the cultural supernatant with rAd5-PEDV-COE virus was harvested and infected into HEK293 cells again. The COE protein was detectable in the supernatant at 48 h of the infected cells by ELISA at a concentration of 23.4 ug/mL (Fig. 1D), whereas COE protein could not be detected in the supernatant of the Ad5 empty vector control and the mock cell control. Meanwhile, the COE protein expression by the rAd5-PEDV-COE infected cells was further confirmed by the anti-PEDV-COE IFA (Fig. 1E). All these results demonstrate the successful generation of a recombinant rAd5-PEDV-COE adenovirus expressing PEDV-COE.

### rAd5-PEDV-COE induces strong PEDV-specific serum IgG/IgA antibodies in mice

**Fig 2:**
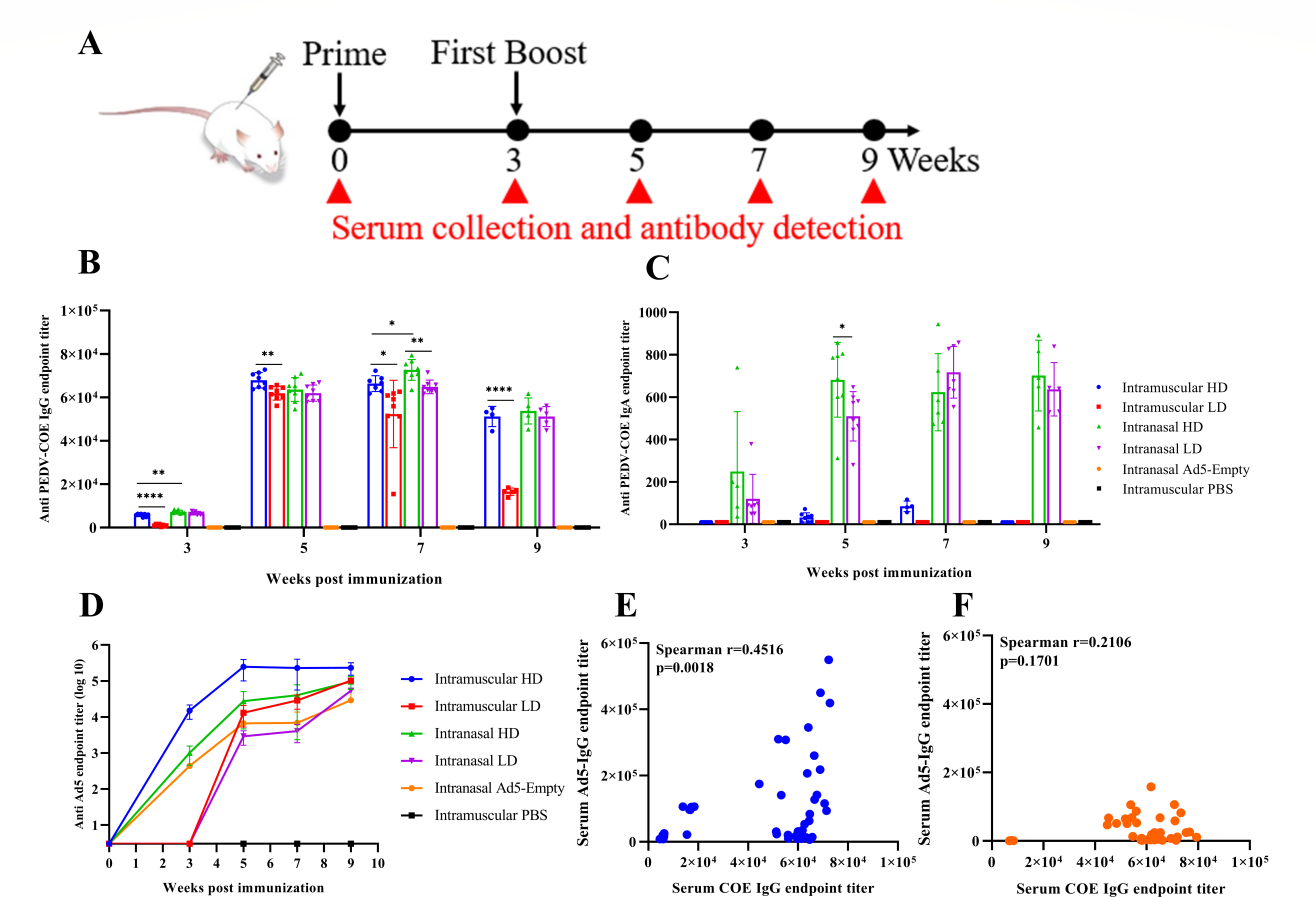
Humoral immune responses of rAd5-PEDV-COE in mice. (**A**) Scheme of immunization and schedule of sampling of sera. (**B**) Anti-COE-specific IgG antibody titers in the serum of mice immunized with rAd5-PEDV-COE. (**C**) Anti-COE-specific IgA antibody titers in the serum of mice immunized with rAd5-PEDV-COE. (**D**) Anti-Ad5-specific IgG antibody titers in the serum of mice immunized with rAd5-PEDV-COE. (**E**) Correlation between COE- and Ad5-specific antibodies in the serum of mice in the IM group. (**F**) Correlation between COE- and Ad5-specific antibodies in the serum of mice in the IN group.

The immunogenicity of the vaccine candidate was evaluated in 6-week-old Balb/c mice. The immunization and specimen collection scheme are shown in Fig. 2A. We collected blood samples at multiple timepoints over a 2-month period and measured serological anti-COE antibody titers by ELISA. The results showed that COE-specific IgG could be induced in the serum of mice in both IM and IN groups after prime immunization and significantly increased after boost immunization. Anti-COE IgG titers in the IN-HD group were significantly higher than those in the IM-HD group at 3 weeks post-prime (*P* < 0.01). IgG titers in the IM group peaked at week two after the boost, whereas the mice in the IN group reached peak antibody titers at week four after the boost, and there was a significant difference in IgG titer between the IN-HD and IM-HD groups at 4 weeks post boost (*P* < 0.05), indicating that a more durable immune response is maintained in the IN group (Fig. 2B), indicating that rAd5-PEDV-COE by IN is more effective at inducing humoral immune responses than intramuscular injection in mice. Systemic IgA was only detected in the serum of mice immunized intranasally, and it was barely detectable in mice immunized intramuscularly even after a booster. IgA titers were maintained at high levels for 6 weeks after the boost immunization. IgA titers in the IN-HD group were significantly higher than those of the IN-LD group at 2 weeks post boost (*P* < 0.05), and there were no significant differences in IgA titers between the two groups at other time points (Fig. 2C). In terms of anti-Ad5 vector-specific antibody responses, we observed that low dosages did not induce antibody responses after prime immunization. Low dose also elicited lower vector antibody responses than high dose after boost immunization, regardless of the immunization route. Interestingly, unlike the anti-COE antibodies, the Ad5-specific vector antibody response induced by intranasal administration was lower than that induced by intramuscular administration (Fig. 2D). Analysis of the correlation between COE-specific IgG and Ad5-specific IgG in serum revealed that these antibodies were positively correlated in the IM groups, whereas there was no correlation in the IN groups (Fig. 2E and F). Taken together, rAd5-PEDV-COE can elicit potent systemic antibody responses in mice.

### rAd5-PEDV-COE induces decent PEDV NAbs in mice

**Fig 3:**
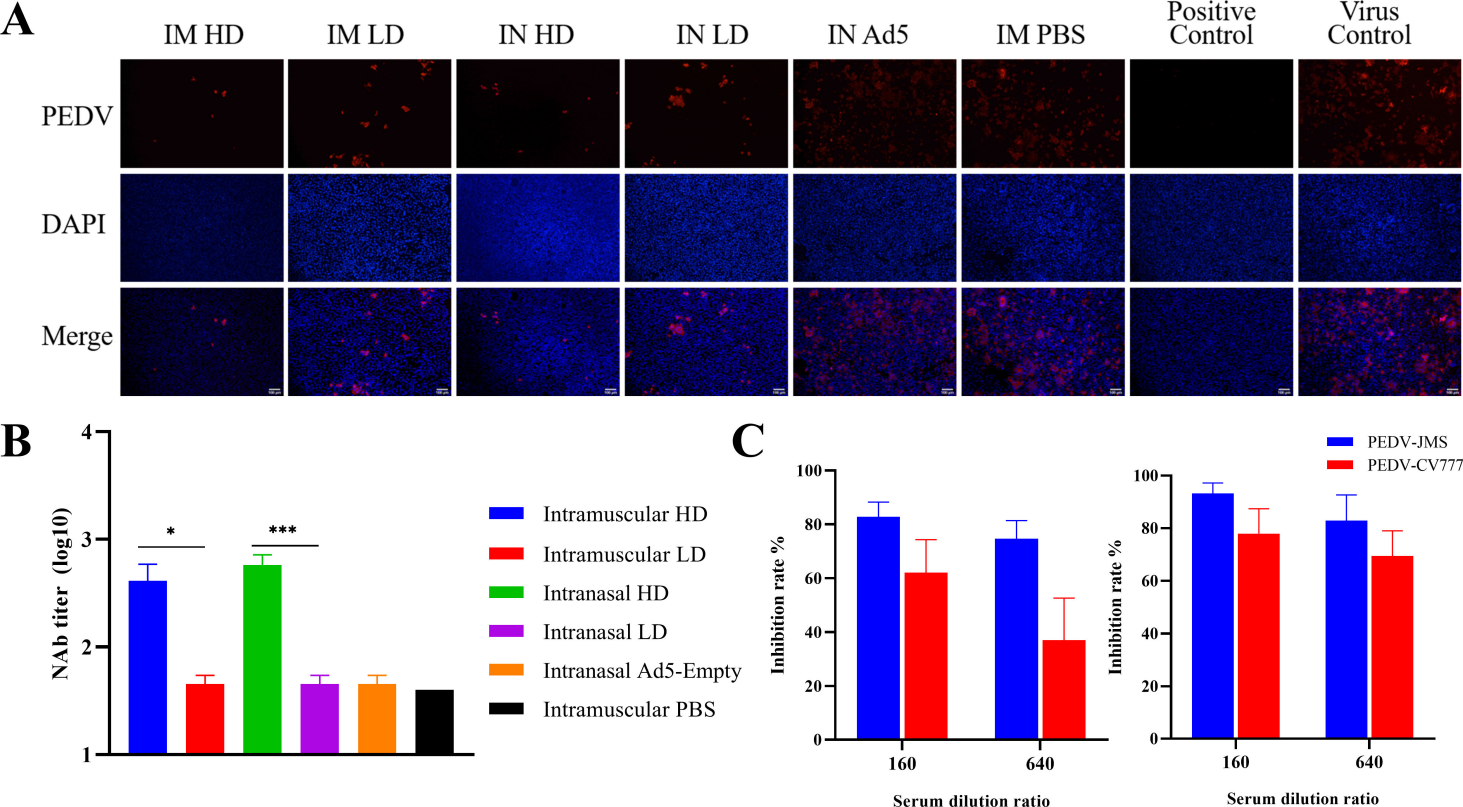
Neutralizing antibody titer in mice immunized with rAd5-PEDV-COE. (**A**). PEDV-infected cells in the neutralization assay were detected by anti-PEDV N immunofluorescence assay (IFA). Anti-PEDV N protein: red; DAPI: blue; Serum dilution: 1:40, Bars = 100 µm. (**B**) Neutralizing antibody titers in sera of mice immunized with rAd5-PEDV-COE against PEDV JMS. (**C**) Neutralizing antibody titers in sera of mice immunized with rAd5-PEDV-COE against PEDV CV777 (left: IM-HD group; right: IN-HD group).

Neutralizing antibodies are crucial in the fight against PEDV infection. Serial neutralizing activity against the PEDV-JMS at 2 weeks post boost immunization was evaluated by immunofluorescent visualization of infected Vero cell monolayers using anti-PEDV N mAb IFA. Typical fluorescent images of infected monolayers are shown in Fig. 3A; the virus-infected cells were significantly decreased after pretreatment with serum of rAd5-PEDV-COE immunized mice. The serum of mice in the IM/IN-HD group showed more neutralizing activity against PEDV infection compared with those of the IM/IN-LD group, with 1:410 and 1:576 NAb titers (Fig. 3B), respectively, which are consistent with the anti-COE-binding antibody results (Fig. 2). Next, we detected whether the serum could broadly neutralize PEDV Genotype one classical strain CV777. The serum from both IM-HD and IN-HD groups was capable of substantially neutralizing PEDV CV777, indicating good crossing protection, although the neutralizing titers against CV777 were significantly less than that of JMS (Fig. 3C). No inhibiting activity was observed in the Ad5 vector and PBS control groups. These data indicate that rAd5-PEDV-COE could elicit potent and broad neutralizing antibodies against PEDV.

### Second boost of rAd5-PEDV-COE could further enhance the immune response

**Fig 4:**
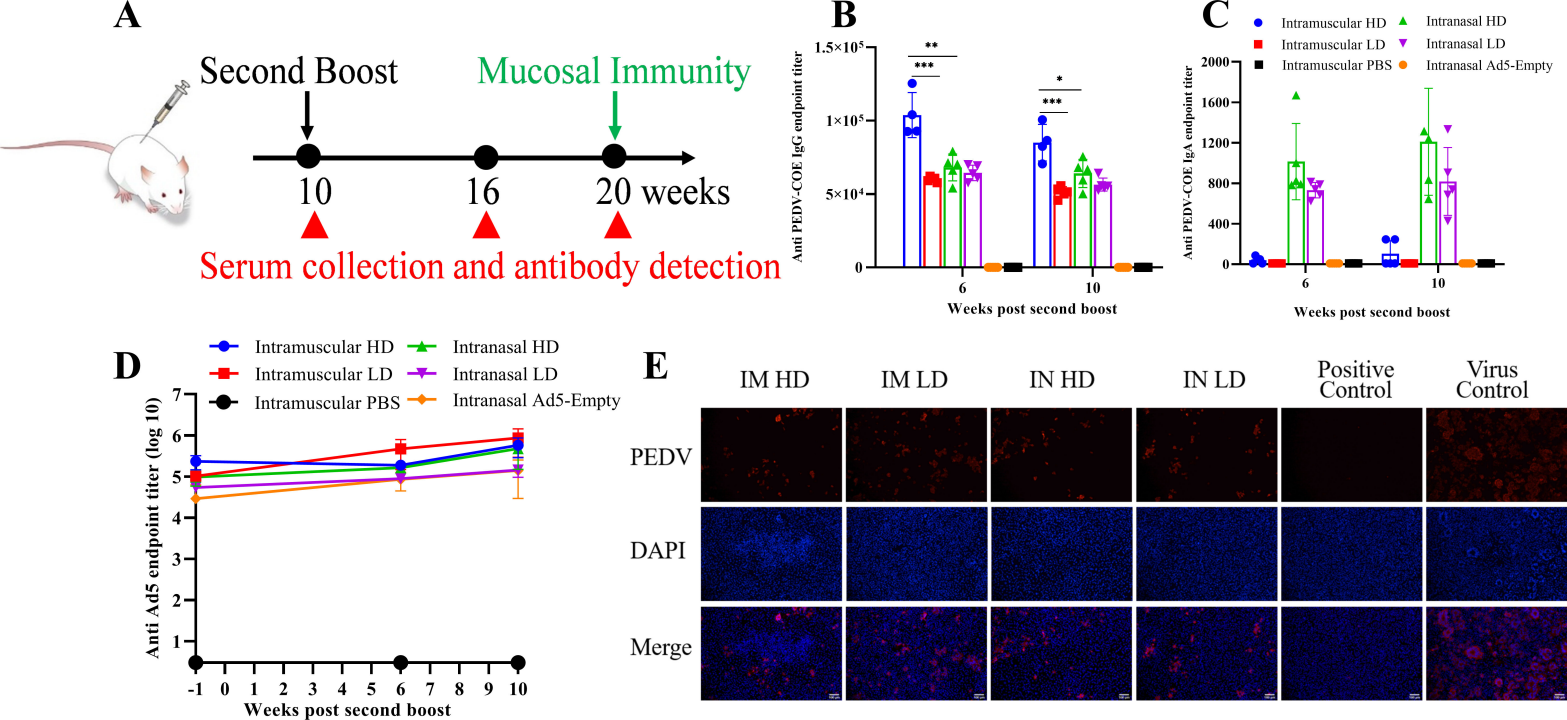
The second booster enhanced the immune response induced by rAd5-PEDV-COE. (**A**) Scheme of booster immunization and schedule of sampling of sera, intestinal contents, and BALF. (**B**) Anti-COE-specific IgG antibody titers in the serum of mice immunized with rAd5-PEDV-COE post-second boost immunization. (**C**) Anti-COE-specific IgA antibody titers in the serum of mice immunized with 5-PEDV-COE post-second boost immunization. (**D**) Neutralizing antibody titers in the serum of mice immunized with rAd5-PEDV-COE post-second boost immunization, Bars = 100 µm. (**E**) Anti-Ad5-specific IgG antibody titers in the serum of mice immunized with rAd5-PEDV-COE post-second boost immunization.

One of the major concerns to Ad5 vector-based vaccines is the interference of preexisting vector immunity. It is interesting to evaluate the boosting efficiency following the twice inoculations of a rAd5-PEDV-COE that has elicited strong vector immunity. To address the question, we performed another boost at 10 weeks post-prime immune to examine whether the antibody titer could be further increased by the second boosting. During the second boost phase, we found that the COE-IgG titer of the IM-HD/LD group further increased significantly at 6 weeks post another boost compared with that at 6 weeks after first boost, with *P*-value of <0.001 and <0.0001, and maintained at a high level at 10 weeks post the last boost. In contrast, the COE-IgG titer of the IN group showed no significant change, and a significant difference was found in the IgG titer between the IM-HD and IN-HD groups post second boost, with *P*-value of <0.01 and <0.05 at 6 and 10 weeks post-second boost, respectively (Fig. 4B). Interestingly, unlike the pattern of anti-PEDV-COE IgG, the second boost resulted in a gradually increased anti-PEDV-COE IgA until 10 weeks post the last boost in both the IN-HD and IN-LD groups (Fig. 4C) although no statistically significant difference was found between the two-time points before and after the second boost, with *P*-value of 0.1388 and 0.1996, respectively. Unlike the enhanced effect of anti-PEDV-COE IgG in the IM group, another boost still failed to induce serum anti-PEDV-COE IgA antibody response in the IM group (Fig. 4C). Ad5 vector-specific IgG antibodies also further increased after the last boost just as the PEDV-COE IgG antibody did (Fig. 4D). Additionally, we detected significant neutralizing activity of the serum at 6 weeks after last boost, indicating the long persistence of neutralizing antibody (Fig. 4E). These results indicate that a third inoculation only provides limited beneficial and is related to the routes of vaccine inoculation.

### PEDV COE-specific IgG in mucosa primarily derives from the peripheral system

**Fig 5:**
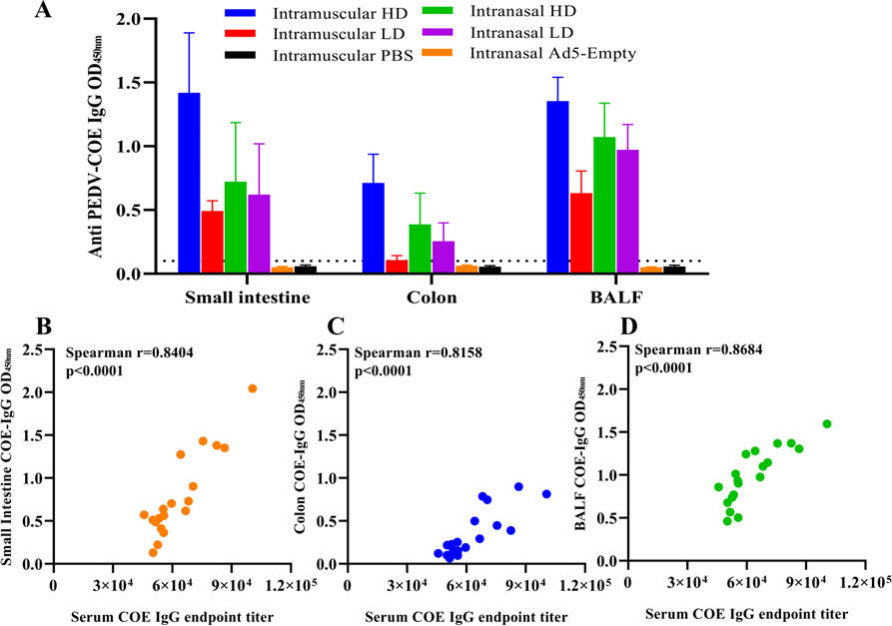
PEDV-COE-specific IgG antibody response in mucosa. (**A**) Anti-COE-specific IgG antibody in the mucosal sample (Intestinal contents and BALF) of mice immunized with rAd5-PEDV-COE. (**B**) Correlations of COE-IgG in serum and COE-IgG in small intestinal contents. (**C**) Correlations of COE-IgG in serum and COE-IgG in colon contents. (**D**) Correlations of COE-IgG in serum and COE-IgG in BALF.

To explore the mucosal immunity by rAd5-PEDV-COE, the COE-IgG antibody of mucosal samples (Intestinal contents and Bronchoalveolar Fluid) was detected by indirect ELISA. The intestinal contents of the mice in the IM-HD group showed better COE-IgG response than those in other groups, so did the colon contents and BALF of the mice in the IM-HD group. No significant difference in the COE-IgG OD values in mucosa between the IN-HD and IN-LD groups was observed, but both were higher than those in the IM-LD group. The mucosal COE-IgG in the small intestines and lungs was higher than that of the colon (Fig. 5A). Correlation of the COE-IgG antibody between mucosal samples and serum were all significantly positive, and the correlation coefficients of COE-IgG in the serum to those of the small intestinal contents, colon contents, and BALF were 0.8404, 0.8158, and 0.8684, respectively (Fig. 5B through D), suggesting that mucosal anti-PEDV IgG is primarily derived from the peripheral system.

### Intranasal delivery of rAd5-PEDV-COE elicits an inferior anti-Ad5 vector immunity in mucosa compared with intramuscular injection

**Fig 6:**
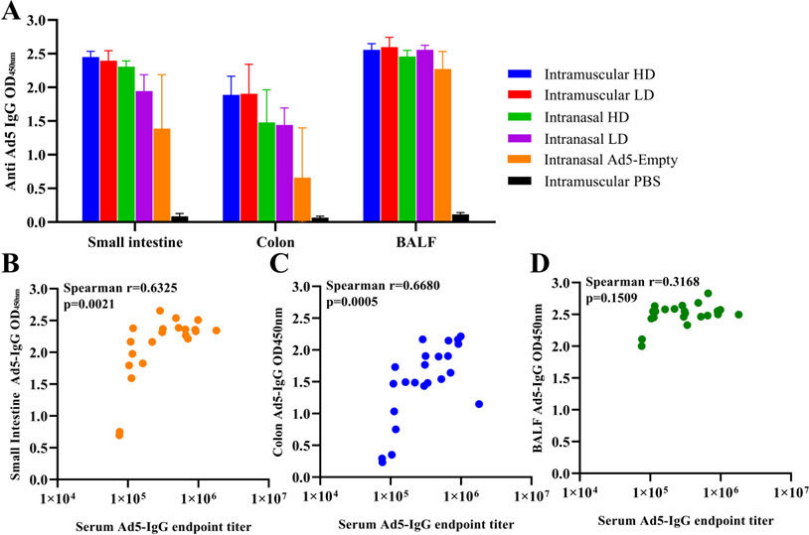
Correlations of Ad5-specific IgG antibodies in serum and mucosa. (**A**) Anti-Ad5-specific IgG antibody in the mucosal sample (intestinal contents and BALF) of mice immunized with rAd5-PEDV-COE. (**B**) Correlations of Ad5-IgG in serum and Ad5-IgG in small intestinal contents. (**C**) Correlations of Ad5-IgG in serum and Ad5-IgG in colon contents. (**D**) Correlations of Ad5-IgG in serum and Ad5-IgG in BALF.

Similarly, intestinal contents and BALF of the mice from 10 weeks after the last boost were collected for detection of Ad5-IgG in the mucosa. We found that high levels of Ad5-IgG were detected in both intestinal contents and BALF, and the Ad5-IgG antibody in BALF was higher than that in intestinal contents. The level of Ad5-IgG antibody in mucosal samples of the mice in the IM group was significantly higher than that in the IN group, suggesting that intranasal delivery of Ad5-based vaccine induced an inferior anti-Ad5 vector immunity compared with intramuscular injection (Fig. 6A). Just as the mucosal anti-PEDV IgG, Ad5-IgG in small intestinal and colon contents was positively related with Ad5-IgG titer in serum, with correlation coefficients of 0.6325 and 0.6680, respectively (Fig. 6B and C), indicating their peripheral system origin. Surprisingly, no correlation between Ad5-IgG values in BALF and Ad5-IgG titer in serum was observed (Fig. 6D).

### IN inoculation of rAd5-PEDV-COE instead of IM elicited strong mucosal immunity

**Fig 7:**
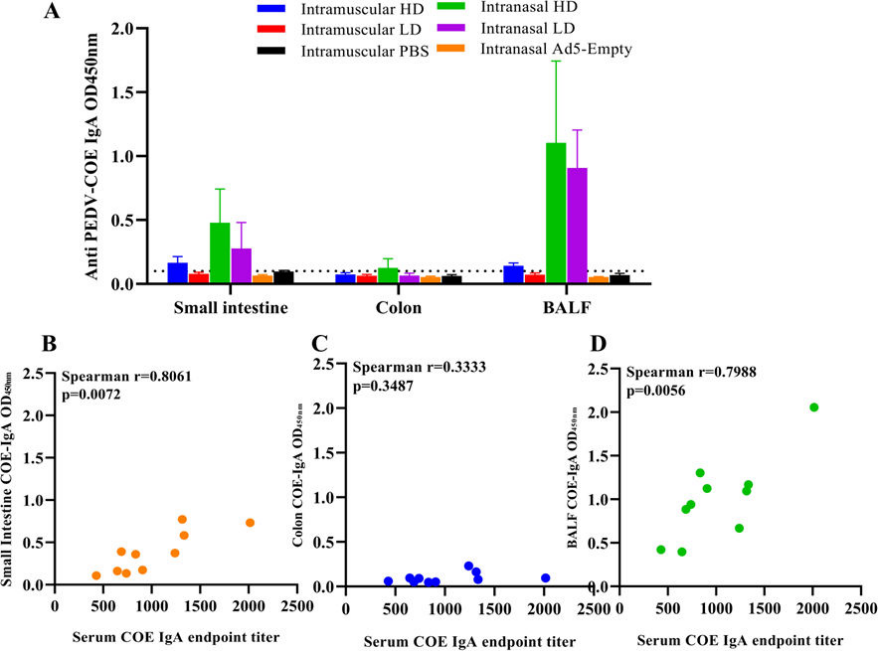
Intranasal delivery of rAd5-PEDV-COE elicits IgA antibody response in mucosa. (**A**) Anti-COE-specific IgA antibody in the mucosal sample (intestinal contents and BALF) of mice immunized with rAd5-PEDV-COE. (**B**) Correlations of COE-IgA in serum and COE-IgA in small intestinal contents. (**C**) Correlations of COE-IgA in serum and COE-IgA in colon contents. (**D**) Correlations of COE-IgA in serum and COE-IgA in BALF.

Mucosal IgA antibody plays substantial roles against gastroenteric viral infection. To evaluate the ability of rAd5-PEDV-COE to induce specific mucosal antibodies, COE-IgA was evaluated in mucosal samples (intestinal contents and BALF) of mice using indirect ELISA. Mice immunized intranasally with rAd5-PEDV-COE showed significantly higher levels of IgA than mice immunized intramuscularly, and the BALF of the IN-HD/LD group showed higher IgA levels than that in small intestinal contents; no IgA antibodies were detected in colon contents. Consistent with the results of serum anti-COE IgA, no anti-COE IgA was detected in all mucosal samples of the IM-HD/LD group (Fig. 7A). Next, the correlation of IgA antibody in serum and mucosal samples was analyzed, and IgA antibody in serum was positively correlated with that in small intestinal contents and BALF, with correlation coefficients of 0.8061 and 0.7988, respectively (Fig. 7B and D).

## DISCUSSION

PEDV is one of the major economic impacted pathogens for the pork industry, and vaccination is the most effective means to prevent and control PEDV (22, 23). Currently, inactivated and attenuated vaccines against PEDV are the most commonly used worldwide (24–26), but these vaccines failed to successfully cease the epidemic of PEDV. There is great need to develop more effective vaccines against PEDV infection. The novel subunit vaccine based on adenovirus vector has been approved to be a suitable strategy to prevent coronavirus-related diseases, such as SARS-COV-2 (27–30). In this study, a recombinant adenovirus expressing COE epitope in S protein of PEDV was constructed, and the immunogenicity of rAd5-PEDV-COE was evaluated in mice with different immunization routes and doses. rAd5-PEDV-COE induced potent systemic humoral response, including functional neutralization antibodies against PEDV through various doses and immunization routes, and IN vaccination of rAd5-PEDV-COE elicited strong respiratory and gastrointestinal mucosal immunity. As for the immune dose, the antibody response induced by different doses is related to the immune routes, and a significant difference could be observed in the antibody titer in the IM-HD and IM-LD groups, whereas no significant difference was observed in the antibody titer in the IN-HD and IN-LD group.

PEDV primarily targets and infects the intestinal epithelial *in vivo* and causes catastrophic injury and damages in the small intestines, thus local mucosal immunity is critical for intestinal homeostasis and is crucial to prevent and control PEDV infection (31). Recently, it is established that airborne transmission through disrupting the respiratory endothelial barrier is another potential important transmission route for PEDV except the established oral–fecal route (32). Therefore, local mucosal immunity in both respiratory airway and intestines would confer protective roles against PEDV infection. However, inactivated and attenuated vaccines are generally less efficient to induce respiratory and gastrointestinal local mucosal immunity after inoculation. In this study, vaccination of rAd5-PEDV-COE could induce significant systemic COE-specific antibody after vaccination with different doses and routes. Interestingly, we demonstrated that only intranasal rAd5-PEDV-COE elicited potent IgA in the intestinal contents and BALF of mice though COE-specific IgG antibodies detected in both mucosal surfaces regardless of being immunized intramuscularly or intranasally. This is in agreement with SARS-COV-2 vaccine results that adenoviral vector ChAdOx1-S vaccine by intranasal vaccination elicited a superior IgA antibody titer in the sera and mucosa compared with the intramuscularly immunized group (33). Notably, unlike the PEDV-COE IgG, intranasal rAd5-PEDV-COE elicited lower levels of serum antibodies against the Ad5 vector itself compared with IM vaccination, and this is not related to the vaccination dose (Fig. 2D). In agreement with this result, the serum PEDV-COE IgG did not correlate with the serum anti-Ad5 IgG in the IN group (Fig. 2E) but exhibits a significant correlation with the serum anti-Ad5 IgG in the IM group (Fig. 2D). These findings suggest that the intranasal rAd5-PEDV-COE as the candidate vaccine is a promising strategy for the control of PEDV pandemic.

Defining the origins of mucosal local antibodies is challenging but necessary to facilitate rational mucosal vaccine design. We found that IN not IM could induce robust antigen-specific IgA in the peripheral system and the mucosa (Fig. 2 and 7). To understand the crosstalk between mucosal sites and peripheral systems, we analyzed the correlation between the mucosal antibodies and serum antibodies against both target antigen and vector adenovirus. Mucosal PEDV-COE IgG from both intestinal content and BALF is strongly correlated with serum PEDV-COE IgG (Fig. 5), and the vector antibody anti-Ad5 IgG did the same (Fig. 6), indicating that intestinal or BALF mucosal IgG primarily derives from the peripheral system. Interestingly, PEDV-COE IgA in the small intestines and not the colon is positively correlated with serum IgA, indicating different origins for different intestine segmental IgA.

Studies have shown that vaccine boost doses are important for restoring vaccine efficacy and further limiting virus circulation, leading to a significant increase in neutralizing immunity and higher levels of antibody titers against variant SARS-COV-2 strain (34). Thus, we performed a second boost vaccination to confirm whether an additional vaccine booster is necessary after prime-boost vaccination. We found that the second intramuscular boost vaccination with rAd5-PEDV-COE could enhance the COE-specific IgG antibody response 2 months after the first boost vaccination, but the second intranasal boost vaccination showed no significantly enhanced COE-specific IgG antibody response, but the IgA titer in the IN-HD group gradually increased after the second boost. These indicate that a second booster using the same vaccine would have a limited benefit. This is consistent with the published results of SARS-CoV-2 vaccination that a hybrid boosting strategy for COVID-19 vaccination confers more effective cross-variant neutralization than the same boosting strategy (35).

### Conclusion

In this study, we comprehensively evaluated the immune response of a vaccine with the Ad5-encoding COE protein in the Balb/c model. The recombinant adenoviral vaccine, rAd5-PEDV-COE, elicited potent humoral and mucosal immune responses by intramuscular or intranasal immunization, especially, intranasal immunization of rAd5-PEDV-COE could stimulate IgA antibody responses in both sera and mucosa of mice. Therefore, intranasal immunization of rAd5-PEDV-COE has the potential to become a promising vaccination strategy against PEDV.
